# The successful implementation of Scotland’s Hepatitis C Action Plan: what can other European stakeholders learn from the experience? A Scottish voluntary sector perspective

**DOI:** 10.1186/1471-2334-14-S6-S7

**Published:** 2014-09-19

**Authors:** Leon Wylie, Sharon Hutchinson, David Liddell, Nicola Rowan

**Affiliations:** 1 Hepatitis Scotland, Glasgow, United Kingdom; 2 Glasgow Caledonian University, and Health Protection Scotland, Glasgow, Scotland, United Kingdom; 3 Scottish Drugs Forum, Glasgow, Scotland, United Kingdom; 4 Health Protection Scotland, Glasgow, Scotland, United Kingdom

## 

In 2004, the Scottish government declared that “Hepatitis C is one of the most serious and significant public health risks of our generation” [1]. Social and political recognition of the scale of the problem, and of its strong links to health inequalities, spurred policy-makers to allocate significant government funding to a nationally coordinated Hepatitis C Action Plan [2], with an evidence-gathering and consultation phase followed by a planning and action phase.

It appears that the successful implementation of the Hepatitis C Action Plan, coupled with an increase in the availability of addiction treatment, has contributed to a significant decline in hepatitis C virus (HCV) incidence, sharp increases in the numbers of people accessing HCV treatment and achieving sustained viral response, and an overall decrease in population prevalence of HCV [3]. The Scottish Action Plan is reflected in many interventions promulgated in ECDC and ECDDMA Guidance: Prevention and Control of Infectious Diseases among People Who Inject Drugs [4] and is a country-level example of evidence-based interventions being effective in practice. This commentary highlights the example set by Scotland in its national approach to hepatitis C and identifies voluntary sector actions and other key elements that contributed to the overall success of the two phases of the action plan.

In 2008–2009, approximately 39,000 people in Scotland were estimated to be chronically infected with HCV, and the overall HCV prevalence rate amongst people who were either currently injecting drugs or had injected drugs in the past stood at about 60% [3]. The identification of HCV as a priority public health risk in Scotland and the development of an effective response were outcomes of a unique collaboration of public health, university and patient representative organisations. This partnership, driven by Health Protection Scotland, used scientific and political pressure to ensure that HCV was addressed in a serious, practical and, importantly, whole-of-country manner.

Voluntary sector organisations, representing people affected by HCV and the services that supported them, were particularly active in raising political awareness of the human impact of the illness and also its links to inequalities. One of those organisations, the UK Hepatitis C Resource Centre (UKHCRC), was the forerunner of Hepatitis Scotland. The UKHCRC ‘s parliamentary lobbying work, alongside the Hepatitis C Trust and other Scottish charities such as the Scottish Drugs Forum, informed the political environment regarding the direct impact the illness has on patient lives and helped generate the cross-party consensus necessary to support subsequent funding and policy initiatives.

During Phase I of the Action Plan, it was estimated that the number of people newly acquiring HCV each year through injecting drug use was between 1,000 and 1,500 [2][5]. With HCV antibody testing only available since 1991 and with positive diagnoses often made many years after infection, trends in HCV incidence in Scotland cannot easily be inferred from currently diagnosed cases [6].The Scottish HCV epidemic may have its roots in the rapid explosion in intravenous drug use across Europe in the early 1980s, with the primary catalyst being an influx of opiates from western Asian sources such as Afghanistan [7].

Although National Health Service (NHS) regional boards were treating a limited number of HCV patients in the early 2000s, modelling by Health Protection Scotland indicated that treatment was not reaching enough people to have a major impact on the growing burden of liver disease [8]. The number of prison inmates being treated for HCV was very low [3], especially considering the high number of incarcerated people who inject drugs and the consequently high HCV prevalence rate in prisons [9].

There was significant variation in the distribution of needles/syringes and other injecting paraphernalia, as well as in the nature of educational and harm reduction interventions offered by needle exchange services across Scotland [10].The provision of opioid substitution treatment (OST) also varied, ranging from the relatively low threshold supply in the Edinburgh region to some rural areas with waiting times of more than 18 months from initial contact to treatment initiation [11].

Key researchers across the United Kingdom were involved in the first “evidence-gathering” phase of the action plan. This built the evidence, systems and infrastructure for Phase II, which introduced 34 high-level strategic actions that aimed to improve prevention, diagnosis, treatment, care and support services using a nationally co-ordinated approach.

An important factor in staging an appropriate public health response is adequate funding. The careful cultivation of political support, based on evidence-gathering and patient-based advocacy, was rewarded with initial Scottish government ring-fenced funding of £4 million for the first phase of the plan, followed by £43 million for the second phase. The use of these funds was tied to strict project targets based around hepatitis testing and treatment and to a stepwise change in the distribution of clean injecting equipment. This new funding was in addition to funds then being used for Hepatitis C treatment by the regional NHS units.

Another key factor in the success of the plan was the project management approach adopted by the decision-makers from the beginning. Under this approach it was acknowledged that it was likely to be most effective to set and attain achievable goals firmly based in evidence-based practice, even if at times these goals were not as extensive as all would wish. The project management approach controlled ongoing costs, as well as gaining very significant cost savings through national collective bargaining for HCV medication and injecting equipment.

Overall coordination, overseen by a national coordinating group, was tasked to three sector working groups and was enhanced by the implementation of local Managed Care Networks (MCNs). An MCN is a multidisciplinary group, including voluntary sector representatives if available, responsible for a health district’s strategic approach. National guidance documents were produced by task-specific working groups led by key experts, using an evidence-led nationally consultative approach. An important outcome of the evidence-gathering phase was the building and refinement of information systems that informed – and continue to inform – many aspects of strategic direction. The expansion in data-gathering capability affords a continuing real-time comparison and analysis of regional incidence, prevalence and treatment-related data trends.

At the national substance use policy level, which falls under the purview of the Scottish Executive Justice Department rather than the Scottish Executive Health Department, increasing concerns about drug-related deaths and poor treatment outcomes for substance users led to recognition that the provision of OST was dependent on which health board area was the treatment provider. A national target for OST waiting time was introduced in 2008; this produced synergies with other preventative measures associated with the Action Plan [12].

The relative success of the Scottish Action Plan, as recent evidence suggests, has provided a working model for other countries to follow [13]. The incidence of HCV infection among people who inject drugs across Scotland was estimated to have been reduced, potentially by as much as half within a matter of a few years [14]. The concomitant increase in HCV treatment initiation (from 468 cases in 2007–2008 to 1,052 cases in 2012–2013) has also contributed to an overall decrease in HCV prevalence in Scotland, to approximately 38,000 cases in 2012 [3]. There has been a large increase in HCV testing in drug services [15], and hence a greater number of patients who access treatment are from marginalised populations. Prison inmates comprise 11% of those treated, up from four percent in 2007–2008 [3].

It is also recognised that this Action Plan would not have been as effective without serious government investment. The Action Plan approach has been of benefit to ever greater numbers of patients, and given the wide variation or lack of effective national policy approaches to HCV across Europe, the lessons learnt by Scotland in harnessing voluntary sector, health and university institutions to drive forward political consensus can be used to further develop a pan-European strategic approach to HCV.

## Competing interests

The authors declare that they have no competing interests.

## Declarations

This article has been published as part of *BMC Infectious Diseases* Volume 14 Supplement 6, 2014: Viral Hepatitis in Europe. The full contents of the supplement are available online at http://www.biomedcentral.com/bmcinfectdis/supplements/14/S6. The publication charges for this supplement were funded by AbbVie as an unrestricted grant to Rigshospitalet, the University of Copenhagen. AbbVie further funded the printing of the supplement with additional financial support from the Drug Prevention and Information Programme (DPIP) of the European Union.
